# Supplementary material for: Understanding antibiotic use in the community setting in Thailand: Does communication matter?
Source: PLoS One. 2024 Apr 2;19(4):e0298972. doi: 10.1371/journal.pone.0298972 (PMC10986969; doi:10.1371/journal.pone.0298972)
Supplement: S1 File — (PDF) [file pone.0298972.s001.pdf]

S1 File- Research tools

Material used in this paper was derived from transcripts including the following questions:

Phase 1.

IDI Suppliers:

*The medicines [QUESTIONS 2-5: * ONLY FOR BUSINESS OWNERS OR IN-CHARGE HEALTH WORKERS]*

1) Please tell me briefly about the range of different medicines that you sell. Which are the popular ones?
2) * What is/are the source/s of the various medicines that you sell? Do your suppliers bring the medicines to your shop, or do you pick them up yourselves from your suppliers?
3) * What informs your decision to buy from the suppliers that you have mentioned? (Probe for details of quality, cost, credit facilities, packaging/box/container, incentives, etc.)
4) * What are your experiences with getting your supply of different medicines? Probe regarding availability/shortages etc., and specifically for antibiotics.
5) * Where do you think the medicines you sell are manufactured? What are your impressions about the quality of the medicines from different origins? Are certain origins more popular than others?
6) Do you feel you know enough about the different medicines that you sell (what they treat, side effects etc.), or are there certain medicines that you would like to know more about? What would be the best way for you to learn about these medicines?
7) Which medicines do you usually sell to people who have coughs and colds? What determines the length of the course that you supply?
8) What do you know about antibiotics? Explain. What proportion of all the medicines that you sell are antibiotics? Which are your five most commonly sold antibiotics?
9) For which illnesses do you usually sell antibiotics? What determines the choice of antibiotic?
10) How and where do you store your antibiotics?
11) Do you check the expiry dates on the antibiotics you sell? What do you do with medicines that have passed their expiry date?
12) Do customers ever bring back unused medicines? If so, which medicines are most commonly brought back; and what do you do with these?

The customers

13) Can you describe how the dispensing process works here, from when a customer arrives to when they leave with their medicines?
14) Do many of your customers ask for particular medicines without any prescription (*either* through self-medication, *or* on verbal recommendation of health workers)? Is there a certain sort of customer who does this (e.g. male, female, young, old), and what are the most common conditions that they treat on this basis? Do they ask specifically for antibiotics? If so, which type of antibiotic?

15) Do you think that medicine sellers in this community ever feel encouraged to sell their customers antibiotics that may not be needed? If so, is this encouragement related to pressure from the customers or from the suppliers? Do the sellers go ahead and sell the medicines in such situations, or not? Details.
16) Do you ever give any sort of information (verbal or written) to your customers about the antibiotics that you sell them? What information do you give? Do they ever ask questions? Examples. *[Probe: do you ever ask about allergies?]*
17) Do your customers ever voice any concerns about the various medicines you sell? Details?
18) Are you aware of any medicine sellers in this area who *either* sell incomplete doses of antibiotics *[probe: if, for example, a customer doesn't have money for the whole dose. Or for any other reason.], or* who sell more antibiotics than are needed *[probe: for future use]?* Are these practices generally seen as acceptable or not? Details.
19) Do you ever dispense a mixture of antibiotics combined with other medicines? If yes, can you give an example of such a mix that you sell?
20) Do you think that your customers sometimes fail to take their full course of antibiotic treatment? If so, why do you think this is?
21) Do you ask your customers if they have used antibiotics before and for what conditions/diseases, in particular when they are requesting a specific antibiotic?

Antibiotic resistance

22) Do you think you have a good understanding of what antibiotic resistance is, how it is caused, and what its implications are? Is there anything on this topic that you would like to know more about? Details.
23) What do you think would be the best way to inform people (both medicine sellers and the community) about proper antibiotic use and the dangers of antibiotic resistance?
24) Overall, what do you think should be done to improve appropriate antibiotic use and decrease antibiotic resistance in this country?

Regulatory issues [ONLY FOR BUSINESS OWNERS OR IN-CHARGE HEALTH WORKERS]*

25) * What are the regulations that you have to follow in order to sell antibiotics [probe regarding prescriptions]? What challenges, if any, do you face in following these regulations?
26) * Do you think that most sellers know the regulations regarding selling antibiotics? If so, do they always follow them? If not, why not? Do you know whether these regulations are being checked or audited by authorities?
27) * Are there any penalties for sellers who sell antibiotics without prescription? Details.
28) * Do you think current government regulations are sufficient to control inappropriate antibiotic use? If not, how could things be improved?
29) * FOR PRIVATE SECTOR SUPPLIERS ONLY: What would happen to your business if antibiotic sales declined due to closer compliance with regulations on dispensing antibiotics? Would this be a problem for you or your business? If yes, how? What would you suggest as a means of resolving this problem?

We have finished the interview. Thank you for your participation.

IDIs community members:

Accessing treatment

1) How would you rate your overall health?
2) What do you do when you have any of these conditions: [Probes: self-medication, health centre, pharmacy, traditional healer etc.]
   a. Fever
   b. Headache
   c. Cough/cold
   d. Diarrhea
   e. Wound or skin infection
   f. Severe illness
   g. A sick child

   Why do you choose these particular healthcare options?
3) How long does it take you to get to the place/s where you usually go for treatment, and how much does it cost to get there (if transport is necessary)? Is this convenient, or would you prefer something easier to reach?
4) How do you cover the costs for treatment when you or a member of your family is unwell? How easy is it for you to cover these costs, and what do you do if you are unable to cover them.
5) What would you do if there is a particular medicine you are seeking but the supplier doesn't have it? (Probe: buy an alternative medicine from the supplier, go somewhere else etc.)
6) Do you ever ask for or use medicines from other people who have similar illnesses to your own? Details (e.g. from whom, which medicines, for which illness, reasons, etc.)

The supplier/seller of medicines

7) Do you usually ask the supplier for a particular medicine, or do you usually let them decide which would be best for the illness you have?
8) Do you usually receive instructions (verbal or written) for using the medicines from the supplier? In general, do you think they are well informed about the medicines they sell, so that you would trust their word and want to follow their instructions? Details. (Probe: by type of suppliers)
9) Do you ever buy less medicine / fewer pills than recommended by your healthcare provider? *[Probe: do you sometimes not have enough money to buy the full course of treatment?]* Details.

The medicines

10) What sort of medicines, if any, do you have at home at the moment, and what are these medicines for?
11) Do you know what antibiotics are, and what sort of conditions they treat? Can you give any examples of antibiotics that you know about?
12) *Now assess the participant's level of knowledge on antibiotics as follows, and register the participant's responses in the separate eCRF. First, pose the 3 multiple choice questions about antibiotics. At each question, ask the participant to select one of the 4 options listed. Second,*

display the first showcard with photos of 3 pills that are commonly available in the study area: paracetamol, a non-steroidal anti-inflammatory medicine, and an antibiotic. Ask the respondent to indicate which is the antibiotic pill according to him/her. Third, display the second showcard with photos of 5 different antibiotic pills that are commonly available in the study area and confirm that these are in fact antibiotics. Ask the participant if he/she visually recognizes any of the antibiotics and, if so, if he/she ever obtained antibiotics. Make sure that the participant's responses are registered in the separate eCRF.

13) *If the participant does not visually recognize any of the antibiotics OR has never obtained antibiotics, skip to question 21. Otherwise, continue with question 13.For each photo ask:* Have you or anyone in your close family ever used this antibiotic? What condition/illness was it used for? Was it effective or not? Do you have a preference for an antibiotic for certain conditions? If so, based on what? Other details.
14) Do you think that antibiotics have any particular benefits or risks? *[Probes: side effects, antibiotic resistance].*
15) If you want to buy antibiotics, what do you do? Do you have to get a prescription from the doctor first, or can you just go directly and buy them at the pharmacy/supplier? *[Probe: can you give more details about the type of supplier you go to, or describe a recent encounter with a supplier?]*
16) Only if you are willing to, you may answer the following questions, but no answer is needed. Are any of the medicines that you have at home now antibiotics? If so, what illnesses were they bought as treatment for?
17) Have you ever bought more antibiotics than you needed at the time, for future use? Details.
18) Do you sometimes stop taking your antibiotics before your pills are finished? Details (how and why). *[Probes: side effects, not sick anymore].*
19) If you ever have unfinished antibiotics, what do you do with them? *[Probes: do you keep them? Do you ever give unused doses to other people who have the same illness you had?]*
20) Are you aware of any special instructions that you need to know about when you take antibiotics? Details.
21) Have you ever heard of antibiotic resistance? Do you know what it is, how it's caused, and what its implications are? Is there anything on this topic that you would like to know more about? Details.
22) Do you think that the medicines you buy are usually of good quality? Have you ever had any bad experiences with medicines because you thought they were not good quality? Details.
23) Do you know whether medicines have an expiry date? If you think so, can / do you usually check the expiry date of the medicines you use? What do you do if the medicines are past their expiry date?
24) In general, where and how do you learn about medicines? If you wanted to know more, where would you go? What would be the best source of information about medicines for you?

We have finished the interview. Thank you for your participation.

If you are interested, you may take along with you this leaflet which contains more information about antibiotics and antibiotic resistance. *Provide printed information in local language*

FGDs community members:

Accessing treatment

1) Please describe the different alternatives that people in this community use to receive treatment if they are ill (i.e. health centre, pharmacy, traditional healer, medicine peddler, etc). What are the main challenges people face in accessing these facilities in terms of mode of transport, time, and cost?
2) In general, how would you describe the *quality* of the health services (public and private, including pharmacies and other medicine suppliers – and referring to staff, equipment, *and* medicines) that serve your community? What, if anything, do you think could be improved?
3) How are the costs of healthcare covered by people in this community? Which are the ways of obtaining medicines, and do you have to pay for the medicines yourself? *[Probes: health insurance, charity, out-of-pocket, other?]*

The medicines

4) Do you think that the medicines available for people in this community are usually of good quality? Have you heard of any bad experiences that people have had with medicines that were caused because they were not good quality? Details.
5) Do you know what antibiotics are, and what sort of conditions they treat? Can you give any examples of antibiotics that are available in this community?
6) *Now display the second showcard with photos of 5 different antibiotic pills that are commonly available in the study area and confirm that these are in fact antibiotics. For each photo ask:* Do people in this community use this antibiotic? What condition/illness is it used for? Is it effective or not? Do people have a preference for an antibiotic for certain conditions? If so, based on what? Other details.
7) Do you think that antibiotics have any particular benefits or risks? *[Probes: side effects, antibiotic resistance].*
8) Under what circumstances do people in this community take antibiotics without a prescription or a without a recommendation from a health care worker?
9) What do you think could be done to ensure the safe use of medicines in this community?
10) Is there any sort of advertising here for medicines, and in particular for antibiotics? If yes, how far do you think this advertising influences people's choices about the medicines they buy?
11) In general, from where do people in this community purchase antibiotics?

The suppliers/seller of medicines

12) Do people usually receive instructions (verbal or written) from the supplier for using the medicines they buy?
13) In general, do you think that suppliers are well informed about the medicines they sell, so that people trust their word and want to follow their instructions? Details. [*Probe specifically for antibiotics*.]
14) Do people ever buy less medicine / fewer pills than recommended by their healthcare provider? IF so, why does this happen? Details. [*Probe specifically for antibiotics*.]

Antibiotic resistance

15) VIGNETTE: A person living in this community had a certain health condition. He went to the hospital and was given some the most appropriate medication for his condition. He took the full course of the medicine but he did not feel better. A few days later he went to another hospital for the same condition and he was given the same medicine that he was given at the first hospital. Once again, he took the full course of the medicine as the doctor instructed and still did not feel better.
    a. What do you call this condition where you take medicines for a condition but the condition does not go away?
    b. Why do you think the medication is not working?
    c. What do you think the person should do?
    d. How do you think the person can be helped?
    e. If someone takes antibiotics for a condition and it does not work, what do you call that condition?
16) What do people do with unfinished doses of antibiotics? Details (how and why).
17) Do people sometimes stop taking their antibiotics before they have finished all the pills they obtained? Details (how and why). [*Probes: do people save them? Do people ever give unused doses to other people who have a similar illness?*]
18) Have you ever heard of antibiotic resistance? Do you know what it is, how it's caused, and what its implications are? [*Question to be adapted according to responses to previous questions, in particular Number 5.*] Is there anything on this topic that you would like to know more about? Details.
19) In general, where and how do people in this community learn about medicines? What do you think would be the best way to inform people in your community about proper antibiotic use and the dangers of antibiotic resistance?

Phase 2.

IDI Suppliers:

1. If you have a customer who is unwell, which three of the following do you consider to be most important when you are interacting with them:
    a) Following the laws of the country
    b) Giving the customer a good price
    c) Providing the best drug for their condition
    d) Making a profit from the transaction
    e) Ensuring that they return
    f) Giving them good health advice
    g) Referring them to another healthcare facility if that is what they need
    h) Other (specify)
2. Have you ever received any sort of training (on the job, or otherwise) about the medicines you sell, and in particular about antibiotics and/or antibiotic resistance? Give details (content, duration, qualification if any, teaching style – i.e. classroom-based,

distance training, teaching via social media/mobile phone app, a combination of different approaches, or other).

3. Do you feel you know enough about antibiotics and antibiotic resistance in order for you to be able to do your job effectively? If not, would you like to know more?
4. If you indicated that you would like to know more in Question 3, is there anything specific about antibiotics and antibiotic resistance that you feel you would like to know more about, or do you think that you need an overall, general training on the topic?
5. If you were to receive training about antibiotics and antibiotic resistance:
    a) What style of training would suit you best (i.e. classroom-based, distance training, teaching via social media/mobile phone app, a combination of different approaches, etc.)?
    b) How many hours per week would you be able to spend on the course?
    c) How much money, if any, would you be willing and able to spend on course fees?
6. When discussing with your customers about the medicines you sell them, do you generally feel that they understand and internalise what you want to tell them? Would you like to receive training on how to improve this communication with your customers?
7. According to you, what is the main difference between antibiotics and pain killers?
8. Do you think that your customers understand the differences between antibiotics and painkillers?
9. We have noticed that many people are confused by the appearance of pills, and they often consider anything in a capsule to be an antibiotic. However, painkillers and other medicines can also come as capsules. Why do you think this confusion exists, and what can we do about it?
10. There are rules and regulations in [country] that govern the sale of medicines, including antibiotics.
    a) Do you personally feel well informed about these rules and regulations?
    b) Do you think that most people who sell or dispense antibiotics in your community know and follow the rules and regulations?
    c) Are you aware of any antibiotic suppliers in your area who have been caught and/or charged for breaking these rules and regulations? Give details.
    d) Would you like to see enforcement of the regulations on the sale of antibiotics enhanced or changed in any way; and if so, how?
11. For which conditions or diseases do you most often dispense or sell antibiotics in your shop/facility? Would you consider it to be useful to have a rapid diagnostic kit for any of these conditions or diseases? If so, which ones?
12. If someone with the following description came to your shop/facility, would you provide them with antibiotics or some other sort of medication? (Yes/no)
    a) 25-year-old otherwise healthy man with diarrhoea for two days (no blood, little pain);

b) 37-year-old lady with pain passing urine, fever 38C, and cloudy smelly urine;
c) 3-year-old with runny nose, fever 38C for one day and crying more than usual. Still eating and drinking fine;
d) 65-year-old diabetic with red, hot, painful swollen leg for three days. The redness has spread from between his toes and on his foot to up to his knee over the three days and he now has loss of appetite;
e) 22-year-old otherwise healthy woman with menstruation and abdominal pain for more than one day, no fever or other symptoms;
f) 53-year-old man with gastric pain for several weeks, no fever.

FGDs community members:

1. Are people in this community aware of the word 'antibiotic', and do you think that most people here know what antibiotics are?
2. Do you think that you would recognise an antibiotic if you saw one? Can you give an example of an antibiotic that is available in your community, and describe what it looks like? Are there any other kinds of drugs that can look similar to antibiotics?
3. There are many different sorts of medicines available in pharmacies and other shops. Among others, these include antibiotics and painkillers. What do you think are the main differences between antibiotics and painkillers, and what are the similarities, if any?
4. What sorts of conditions or diseases might people want to use antibiotics for, and which other conditions or diseases for painkillers? And when would you take both, if ever?
5. What sort of instructions and information do people in your community receive from the suppliers of their medicines (i.e. oral, hand-written, printed, etc.)? Do you think that people in your community generally trust and follow these instructions and information? Yes/no, give details as appropriate.
6. What or who do you think would be a reliable, convenient, and trusted source of information and advice about medicines and antibiotics for you and others in your community?
7. Is there anything about (a) medicines in general and (b) antibiotics specifically that you think people need to understand better in order to take them safely? Yes/no, give details as appropriate.
8. What do you think would be the best and most effective channel/s for people to receive information (i.e. through the radio, by text message, social media etc)?
   a) Which radio stations/newspapers etc do you think would be the best for transmitting messages? Explain why.
   b) Do people in your community use social media for obtaining health information of any sort? If so, details (for informing possible AB information provision).
