# Supplementary material for: Understanding antibiotic use in the community setting in Thailand: Does communication matter?
Source: PLoS One. 2024 Apr 2;19(4):e0298972. doi: 10.1371/journal.pone.0298972 (PMC10986969; doi:10.1371/journal.pone.0298972)
Supplement: S2 File — (PDF) [file pone.0298972.s002.pdf]

S2 File. Healthcare delivery system in Thailand

There are three tiers of healthcare delivery in Thailand: primary, secondary, and tertiary. Primary care units, exemplified by sub-district health promoting hospitals, focus on providing basic curative care, health promotion, and preventive services. Secondary care, represented by community (district) hospitals, general (provincial), and regional hospitals, emphasizes curative care with varying degrees of specialization. Tertiary care, found in university hospitals and large private hospitals, offers the most specialized services [1-3]
The formal healthcare delivery system in Thailand involves both public and private actors, with public facilities serving approximately three-fourths of the population. The public sector encompasses facilities under the Ministry of Public Health and state organizations such as universities and state enterprises. Private sector outlets include hospitals, polyclinics, and clinics [1-3]. In addition to the formal healthcare system, Thailand also features numerous informal healthcare providers. These informal providers comprise pharmacies, drugstores, grocery stores, and other alternative care sources, including traditional healers. Notably, community-based grocery stores in Thailand may offer the non-prescribed poly-pharmaceutical pack, known as Yaa Chud, containing unidentified medicines for consumers[4]

References

1. Kespichayawattana J, Jitapunkul S. Health and health care system for older persons. Ageing international. 2008;33:28-49.

2. Organization WH. The Kingdom of Thailand health system review. Health systems in transition. 2015;5(5).

3. Sakunphanit T. Universal health care coverage through pluralistic approaches: experience from Thailand. Bangkok, ILO Subregional Office for East Asia. 2006.

4. Sunpuwan M, Punpuing S, Jaruruengpaisan W, Kinsman J, Wertheim H. What is in the drug packet?: access and use of non-prescribed poly-pharmaceutical packs (Yaa Chud) in the community in Thailand. BMC public health. 2019;19(1):971. doi: 10.1186/s12889-019-7300-
