# Supplementary material for: Understanding antibiotic use in the community setting in Thailand: Does communication matter?
Source: PLoS One. 2024 Apr 2;19(4):e0298972. doi: 10.1371/journal.pone.0298972 (PMC10986969; doi:10.1371/journal.pone.0298972)
Supplement: Attachment — Submitted filename: Manuscript comments reviewer.pdf [file pone.0298972.s003.pdf]

PLOS ONE

Understanding antibiotic use in the community setting in Thailand: Does communication matter?

--Manuscript Draft--

**Manuscript Number:**	PONE-D-23-33609
**Article Type:**	Research Article
**Full Title:**	Understanding antibiotic use in the community setting in Thailand: Does communication matter?
**Short Title:**	Understanding antibiotic use in the community and communication
**Corresponding Author:**	Malee Sunpuwan, Ph.D<br>Mahidol University Institute for Population and Social Research<br>phutthamonthon, Nakhon Pathom THAILAND
**Keywords:**	Antibiotic use; community setting; communication; Thailand
**Abstract:**	Background : It is known that misuse and overuse of antimicrobials consequently lead to antimicrobial resistance (AMR). Effective communication between dispensers and users is crucial in reducing inappropriate antibiotic use.<br>Objective:This study aims to gain a deeper understanding of the use of antibiotics in the community and seek potential implementation strategies to change dispensers' and users' practices in communication aspects.<br>Methods : Qualitative methods were employed, including in-depth interviews with 18 drug suppliers and 16 community members and eight focus group discussions with key informants. Data were collected in the Kanchanaburi Demographic Health Surveillance System in urban and semi-urban communities in the western region of Thailand. The thematic analysis included communication quality, communication and imbalanced power and beliefs and instruction. Open Code qualitative software program was employed.<br>Results : The study revealed that the quality of communication was significantly influenced by the interaction of antibiotic dispensing with language and information. This interaction created communication constraints between those dispensing antibiotics and the recipients, resulting in a less-than-optimal exchange of information. Consequently, users received limited information concerning the proper use of antibiotics. Furthermore, power imbalances and communication dynamics were perpetuated, mainly stemming from varying levels of access to and knowledge about antibiotics. This imbalance in power dynamics became evident between those dispensing antibiotics and the users. Users, as well as dispensers lacking proper qualifications, found themselves in a precarious position due to their inadequate knowledge of antibiotics. Moreover, it is noteworthy that beliefs often conflicted with antibiotic instructions, leading to challenges in adhering to antibiotic regimens. These challenges primarily arose from misconceptions about antibiotics and concerns about potential side effects, particularly when users started feeling better.<br>Conclusions : The findings highlight the importance of enhancing communication between dispensers and users through future interventions. These interventions should aim to bolster users' understanding of antibiotics and provide clear, authoritative instructions for their proper usage. Investigating innovative communication methods, such as the use of QR codes, presents a promising avenue for consideration. By addressing these communication gaps, we can advocate for the appropriate utilization of antibiotics and mitigate the prevalence of antimicrobial resistance (AMR).
**Order of Authors:**	Malee Sunpuwan, Ph.D
	Sureeporn Punpuing, Ph.D.
	Wipaporn Jaruruengpaisan, MA
	Heiman Wertheim, M.D.
**Additional Information:**	
**Question**	**Response**

Powered by Editorial Manager® and ProduXion Manager® from Aries Systems Corporation

**Financial Disclosure**<br><br>Enter a financial disclosure statement that describes the sources of funding for the work included in this submission. Review the submission guidelines for detailed requirements. View published research articles from *PLOS ONE* for specific examples.<br><br>This statement is required for submission and **will appear in the published article** if the submission is accepted. Please make sure it is accurate.<br><br>**Funded studies**<br>Enter a statement with the following details:<br>• Initials of the authors who received each award<br>• Grant numbers awarded to each author<br>• The full name of each funder<br>• URL of each funder website<br>• Did the sponsors or funders play any role in the study design, data collection and analysis, decision to publish, or preparation of the manuscript?<br><br>Did you receive funding for this work?	Yes
Please add funding details.<br>as follow-up to "**Financial Disclosure**<br><br>Enter a financial disclosure statement that describes the sources of funding for the work included in this submission. Review the submission guidelines for detailed requirements. View published research articles from *PLOS ONE* for specific examples.<br><br>This statement is required for submission and **will appear in the published article** if the submission is accepted. Please make sure it is accurate.	1.The Wellcome Trust , UK<br>2. The Institute for Population and Social Research, Mahidol University, Thailand

**Funded studies**<br>Enter a statement with the following details:<br>• Initials of the authors who received each award<br>• Grant numbers awarded to each author<br>• The full name of each funder<br>• URL of each funder website<br>• Did the sponsors or funders play any role in the study design, data collection and analysis, decision to publish, or preparation of the manuscript?<br><br>Did you receive funding for this work?"	
Please select the country of your main research funder (please select carefully as in some cases this is used in fee calculation).<br>as follow-up to "**Financial Disclosure**<br><br>Enter a financial disclosure statement that describes the sources of funding for the work included in this submission. Review the submission guidelines for detailed requirements. View published research articles from *PLOS ONE* for specific examples.<br><br>This statement is required for submission and **will appear in the published article** if the submission is accepted. Please make sure it is accurate.<br><br>**Funded studies**<br>Enter a statement with the following details:<br>• Initials of the authors who received each award<br>• Grant numbers awarded to each author<br>• The full name of each funder<br>• URL of each funder website<br>• Did the sponsors or funders play any role in the study design, data collection and analysis, decision to publish, or preparation of the manuscript?<br><br>Did you receive funding for this work?"	THAILAND - TH
**Competing Interests**	The authors have declared that no competing interests exist.

Use the instructions below to enter a competing interest statement for this submission. On behalf of all authors, disclose any competing interests that could be perceived to bias this work—acknowledging all financial support and any other relevant financial or non-financial competing interests.<br><br>This statement is required for submission and **will appear in the published article** if the submission is accepted. Please make sure it is accurate and that any funding sources listed in your Funding Information later in the submission form are also declared in your Financial Disclosure statement.<br><br>View published research articles from *PLOS ONE* for specific examples.<br><br>**NO authors have competing interests**<br><br>Enter: *The authors have declared that no competing interests exist.*<br><br>**Authors with competing interests**<br><br>Enter competing interest details beginning with this statement:<br><br>*I have read the journal's policy and the authors of this manuscript have the following competing interests: [insert competing interests here]*<br><br>* typeset	
**Ethics Statement**<br><br>Enter an ethics statement for this submission. This statement is required if the study involved:<br><br>• Human participants<br>• Human specimens or tissue<br>• Vertebrate animals or cephalopods<br>• Vertebrate embryos or tissues	The ethical approval was obtained from the Institute for Population and Social Research Institutional Review Board, Mahidol University, with COA. No. 2016/04-035, and the University of Oxford Tropical Research Ethics Committee, OxTREC: 31-15.

• Field research<br><br>Write "N/A" if the submission does not require an ethics statement.<br><br>General guidance is provided below. Consult the submission guidelines for detailed instructions. **Make sure that all information entered here is included in the Methods section of the manuscript.**	

Powered by Editorial Manager® and ProduXion Manager® from Aries Systems Corporation

**Format for specific study types**<br><br>**Human Subject Research (involving human participants and/or tissue)**<br>• Give the name of the institutional review board or ethics committee that approved the study<br>• Include the approval number and/or a statement indicating approval of this research<br>• Indicate the form of consent obtained (written/oral) or the reason that consent was not obtained (e.g. the data were analyzed anonymously)<br><br>**Animal Research (involving vertebrate animals, embryos or tissues)**<br>• Provide the name of the Institutional Animal Care and Use Committee (IACUC) or other relevant ethics board that reviewed the study protocol, and indicate whether they approved this research or granted a formal waiver of ethical approval<br>• Include an approval number if one was obtained<br>• If the study involved *non-human primates*, add *additional details* about animal welfare and steps taken to ameliorate suffering<br>• If anesthesia, euthanasia, or any kind of animal sacrifice is part of the study, include briefly which substances and/or methods were applied<br><br>**Field Research**<br><br>Include the following details if this study involves the collection of plant, animal, or other materials from a natural setting:<br>• Field permit number<br>• Name of the institution or relevant body that granted permission	
**Data Availability**<br><br>Authors are required to make all data underlying the findings described fully available, without restriction, and from the time of publication. PLOS allows rare exceptions to address legal and ethical concerns. See the PLOS Data Policy and FAQ for detailed information.	Yes - all data are fully available without restriction

A Data Availability Statement describing where the data can be found is required at submission. Your answers to this question constitute the Data Availability Statement and **will be published in the article**, if accepted.<br><br>**Important:** Stating 'data available on request from the author' is not sufficient. If your data are only available upon request, select 'No' for the first question and explain your exceptional situation in the text box.<br><br>Do the authors confirm that all data underlying the findings described in their manuscript are fully available without restriction?	
**Describe where the data may be found in full sentences. If you are copying our sample text, replace any instances of XXX with the appropriate details.**<br><br>• If the data are **held or will be held in a public repository**, include URLs, accession numbers or DOIs. If this information will only be available after acceptance, indicate this by ticking the box below. For example: *All XXX files are available from the XXX database (accession number(s) XXX, XXX.).*<br>• If the data are all contained **within the manuscript and/or Supporting Information files**, enter the following: *All relevant data are within the manuscript and its Supporting Information files.*<br>• If neither of these applies but you are able to provide **details of access elsewhere**, with or without limitations, please do so. For example:<br><br>*Data cannot be shared publicly because of [XXX]. Data are available from the XXX Institutional Data Access / Ethics Committee (contact via XXX) for researchers who meet the criteria for access to confidential data.*<br><br>*The data underlying the results presented in the study are available from (include the name of the third party*	All relevant data are within the manuscript and its Supporting Information files.

and contact information or URL).<br>• This text is appropriate if the data are owned by a third party and authors do not have permission to share the data.<br><br>* typeset	
Additional data availability information:	

Powered by Editorial Manager® and ProduXion Manager® from Aries Systems Corporation

Manuscript added dmy Click here to access/download;Manuscript;Manucsript-1.docx

Understanding antibiotic use in the community setting in Thailand: Does communication matter?

Malee Sunpuwan[1*], Sureeporn Punpuing[1], Wipaporn Jaruruengpaisan[1*] and Heiman Wertheim[2]

[1] Institute for Population and Social Research, Mahidol University, Thailand.

[2] Department of Clinical Microbiology and the Radboud Center for Infectious Diseases, Radboudumc, Nijmegen, Netherlands.

Corresponding author *

E-mail: malee.sun@mahidol.ac.th

E-mail: wipaporn.jau@mahidol.ac.th

Abstract

Background: It is known that misuse and overuse of antimicrobials consequently lead to antimicrobial resistance (AMR). Effective communication between dispensers and users is crucial in reducing inappropriate antibiotic use.

Objective:This study aims to gain a deeper understanding of the use of antibiotics in the community and seek potential implementation strategies to change dispensers' and users' practices in communication aspects.

Methods: Qualitative methods were employed, including in-depth interviews with 18 drug suppliers and 16 community members and eight focus group discussions with key informants. Data were collected in the Kanchanaburi Demographic Health Surveillance System in urban and semi-urban communities in the western region of Thailand. The thematic analysis included communication quality, communication and imbalanced power and beliefs and instruction. Open Code qualitative software program was employed.

Results: The study revealed that the quality of communication was significantly influenced by the interaction of antibiotic dispensing with language and information. This interaction created communication constraints between those dispensing antibiotics and the recipients, resulting in a less-than-optimal exchange of information. Consequently, users received limited information concerning the proper use of antibiotics. Furthermore, power imbalances and communication dynamics were perpetuated, mainly stemming from varying levels of access to and knowledge about antibiotics. This imbalance in power dynamics became evident between those dispensing antibiotics and the users. Users, as well as dispensers lacking proper qualifications, found themselves in a precarious position due to their inadequate knowledge of antibiotics. Moreover, it is noteworthy that beliefs often conflicted with antibiotic instructions, leading to challenges in adhering to antibiotic

regimens. These challenges primarily arose from misconceptions about antibiotics and concerns about potential side effects, particularly when users started feeling better.

Conclusions: The findings highlight the importance of enhancing communication between dispensers and users through future interventions. These interventions should aim to bolster users' understanding of antibiotics and provide clear, authoritative instructions for their proper usage. Investigating innovative communication methods, such as the use of QR codes, presents a promising avenue for consideration. By addressing these communication gaps, we can advocate for the appropriate utilization of antibiotics and mitigate the prevalence of antimicrobial resistance (AMR).

I would suggest that you change or remove ”authoritative” here

Introduction

The threat of antimicrobial resistance (AMR) to public health is of utmost concern, considering the diminishing effectiveness of antibiotics worldwide [1]. Thailand, like many other countries, is not exempt from this threat [2, 3]. In response, the Thai government has initiated various policies and programs aimed at improving antibiotic prescribing practices, increasing public awareness of appropriate antibiotic use, and understanding AMR [3-5]. Notably, the "Antibiotics Smart Use (ASU) program" launched in 2017, with the goal of reshaping antibiotic prescription practices and promoting responsible antibiotic use. Community education campaigns, utilizing diverse educational resources such as infographic posters and pamphlets, have been deployed to educate healthcare professionals and the general public [4, 5]. These concerted efforts have demonstrated promising results in curbing unnecessary antibiotic use [6] and elevating awareness levels [7]. Furthermore, national surveys tracking public knowledge and awareness of AMR and proper antibiotic use have indicated incremental improvements [8]. Nevertheless, antibiotic misuse is still challenging.

The challenge of antibiotic misuse, resulting from a complex interplay of factors involving policymakers, healthcare professionals, and the general public, underscores the gravity of this issue [9]. These factors are intricately linked to variations in governance, drug regulations, pharmaceutical market structures, and cultural influences, all of which contribute to the misuse of antibiotics [10, 11]. Furthermore, a lack of knowledge among healthcare professionals, defensive medical practices, suboptimal patient communication, and time constraints during consultations significantly contribute to the inappropriate dispensing of antibiotics [12-15]. User behaviour, marked by both misuse and overuse, is heavily influenced by social norms and deeply ingrained beliefs [16-20].

Effective communication is widely recognized as pivotal in healthcare to ensure accurate understanding and successful treatment [21]. The global focus on antibiotic

dispensing, which hinges on communication between dispensers and users, has intensified due to its connection with the development of AMR [22-24]. This connection is rooted in the inadequate emphasis on completing the full antibiotic course [25, 26], often caused by insufficient consultation time and ineffective communication skills [27-29], as well as an imbalance in the power dynamic between healthcare providers and clients, leading to premature cessation of antibiotic use [27, 30].

User beliefs about antibiotics often conflict with provided instructions, significantly influencing adherence to treatment plans. These beliefs often stem from misunderstandings, fear of side effects, and a sense of improvement in health [31]. Misunderstandings manifest when users overestimate their knowledge or lack thereof, leading to antibiotic misuse, such as the belief that antibiotics can effectively treat non-bacterial ailments like coughs, colds, body pain, and the flu [32-36]. Fear of side effects and premature discontinuation of antibiotics due to a sense of improvement despite contradictory instructions also influence user behaviour [37, 38].

A systematic review of communication interventions aimed at improving antibiotic use among the public highlighted a dearth of research conducted in Thailand [39]. Among the existing studies, a critical discovery was that improved communication regarding antibiotic knowledge between public health officers and grocery shop owners could reduce the inappropriate sale and use of antibiotics in the community [40]. Another study in Northern Thailand underscored the role of educational activities and local language in enhancing antibiotic attitudes and practices [41].

While studies on communication between health professionals and patients about medications have been a focus for decades [42, 43], limited research in Thailand has encompassed both formal and informal dispensers, as well as users, within the same study. Therefore, this study seeks to comprehensively examine the broader spectrum of dispensers

and users, which includes both formal and informal dispensers. It aims to shed light on the comprehensive examination of antibiotic practices and communication within community settings in developing countries like Thailand.

Methods

The study site

This paper reports on results from the study conducted at the Kanchanaburi Health and Demographic Surveillance System (HDSS) in Thailand, which is one of the INDEPTH (International Network for the Demographic Evaluation of Populations and Their Health in Developing Countries) community-based study sites. It is a sub-study of the project on "Community-level antibiotic access and use in low- and middle-income countries: Finding targets for social interventions to improve rational antimicrobial use", known as the ABACUS (AntiBiotic ACcess and USe) study. Its focus is on appropriate access and use of antibiotics between six different health and demographic surveillance sites in low- and middle-income countries in Asia (Bangladesh, Vietnam, and Thailand) and Africa (Mozambique, Ghana and South Africa) [44]. For more details, see https://abacus-project.org/project/.

The Kanchanaburi HDSS is located in western Thailand. It was established in the year 2000 with a cohort size of approximately 43,000 populations in 2004. This study, however, included about 8,000 persons residing in urban and semi-urban areas of the Kanchanaburi HDSS[45]. Two phases of data collection were taken place in urban and semi-urban communities of the Kanchanaburi HDSS during July 21, 2016 to June 19, 2017 for the first phase and August 26 to September 15, 2018 for the last phase.

Data collection techniques

This paper presents the study's qualitative findings, which involved two phases of data collection. During the first phase, 34 in-depth interviews (IDI) were conducted, involving 18 drug suppliers and 16 community members who use antibiotics. Additionally, six focus group

discussions (FGDs) were held with community members. In the second phase, we conducted follow-up IDIs with eight drug suppliers and two more FGDs with community members, addressing important issues identified in the initial phase. The interview and discussion guides are available in S1 File. The data collection for the IDIs and FGDs was carried out by researchers MS, SP, and WJ, who have extensive training and experience in qualitative research. They have been actively involved in the Kanchanaburi HDSS since 2000. The core questions used in this study were standardized across all the other INDEPTH ABACUS study sites

Sample selection

In-depth interviews (IDIs)

The key informants for IDI were heads or main responsible persons for drug dispensing and community members located in the Kanchanaburi HDSS. In this study, the key informants for antibiotic dispensers (suppliers) were those who worked at both public and private health facilities. There were three healthcare service levels for drug suppliers: tertiary, secondary, and primary (more details in S2 File).

Drug supplier

At the tertiary level, there was one public provincial hospital located in our study area which was willing to participate in this study for the two phases of data collection. Two health facilities were recruited to our study for the secondary level of health care service for the first and second phases. There were three private hospitals located in our study area. Two private hospitals agreed to participate in the first phase, and only one private hospital was left for the second data collection.

The primary level includes health care services in the public and private sectors. The public sector included community health promotion hospitals (CHP) and local health centers. The private sector had private clinics and drug stores. There were 12 CHPs and local health

centers in our study area. According to our qualitative research methodology designed in addition to budget and time constraints, three key informants from the CHP were interviewed in-depth in the first phase. Criteria to select the CHP based on a number of coverage population under each CHP. Therefore, the coverage population's first, second and third rank were selected.   Two CHP participated in the second data collection phase.

The researchers approached private clinics through local knowledge and our networks by providing relevant research information to the owner. The Kanchanaburi provincial health office reported that there were 101 private clinics operated by different level of health profession in our study area. The selection criteria of private clinics based on willingness of private clinic owner. The research team was able to recruit two private clinics in the first phase.

The health provincial office provided a full list of licensed pharmacy shops in our study, which comprised 96 shops. A health provincial officer –a pharmacist- contacted drugstore owners because of participants' trust in local authorities. Finally, six drugstore owners agreed to participate in the first data collection phase. One drugstore was selected for IDI for the second phase because it had the highest number of antibiotic encounters. The owners or pharmacists were the IDI key informant.

Although only medical doctors or licensed pharmacists can dispense antibiotics, some local grocery stores sell drugs and antibiotics by laypersons. Based on community leaders' and village health volunteers' networks, two grocery shops were willing to be in-depth interviewed in the first phase, but not in the second phase.

In this study, the researcher did not substitute the declined health facilities (private hospital, CHP hospital, private clinic, and drug store) because we would like to follow up with the interesting issues that came up during the first phase.

Community members

Another group of key informants for the IDI was community members, who are antibiotic users. The sample selection was based on a simple random sampling technique. A sampling frame was employed from two sources of data, the first source was a list of household members in the Kanchanaburi HDSS database, and the second source was a list of clients that received antibiotics from a health-promoting hospital during the year before the IDI. The population in the sampling frame were stratified into 5 groups – population age 18-59 years (male and female), population aged 60 years or older (male and female), and mothers of children aged 5 years or younger. The researchers employed the SPSS simple random command to select key informants randomly. The 16 community key informants consisted of two males and two females aged 18 to 59 years, two males and two females aged 60 years or older, and eight mothers or guardians of children under five years. The IDI data collection from community members was done only in the first phase.

The gathering information included experience with obtaining and using medicines by community members, focusing on where medicines are obtained, how they are used, and for what reasons. In addition, the researcher collected information related to the issues of accessing treatment, supplier/sellers of medicines, and medicines.

Focus groups discussions (FGDs)

Eight Focus Group Discussions (FGDs) were conducted and divided into two phases. In the first phase, there were six FGDs, while the second phase involved two FGDs. Each FGD consisted of six to eight participants recruited through the community network in the study areas, which were selected based on the population of the Kanchanaburi Health and Demographic Surveillance System (HDSS).

In the first phase, the first four FGDs included two groups of males and two groups of females, with participants aged 18 to 29 years (comprising two FGDs) and those aged 30

years or older (comprising two FGDs). The other two FGDs included village health volunteers and the elderly.

In the second phase of the study, we focused on common themes that emerged from the first phase. It was determined that two specific groups of participants played a crucial role in shaping these themes: males aged 60 or older and females aged 18 to 59 years.

The standardized discussion guideline for the FGD had been used in all sites of the INDEPTH ABACUS study (see S1 File). These core questions aimed to gather information on accessing treatment, medicines, suppliers/sellers, and antibiotic resistance.

Study procedures

The convenient places and times for the participants were the most important issues for the researchers to conduct IDI and FGD. MS, SP, and WJ conducted all IDIs. MS moderated all the FGDs with assistance from SP and WJ. They used the Thai dialect to run IDI and FGD with discussion guidelines encouraging dialogue with optional probes. The IDI lasted 45-60 minutes, while the FGD took 60-90 minutes. An audio record was used for each IDI and FGD if participants consented. One participant in our study did not permit audio-recording the IDI but alternatively gave consent for note-taking.

Data analysis

Audio transcription was done in order to prepare for data analysis. Performing line-by-line coding by using the Open Code program [46]. Subsequently, thematic analysis and codes were assigned to the material. MS and SP determined the thematic saturation. This study applied the 32-item Consolidated Criteria for Reporting Qualitative Studies (COREQ) checklist [47].

Ethical issues

The researchers obtained written and verbal consent from all participants. Someone on the reserve list replaced those participants who decided to decline to participate. The

researchers compensated the participants with a small souvenir (cloth bag). The ethical approval was obtained from the Institute for Population and Social Research Institutional Review Board, Mahidol University, with COA. No. 2016/04-035, and the University of Oxford Tropical Research Ethics Committee, OxTREC: 31-15.

Characteristics of sample

There were a total of 88 individuals who participated in the FGD and IDI, including community members and suppliers. Community members included 30 males and 40 females. Most had completed primary school and were aged between 18 and 82 years. Eighteen suppliers participated in this study, including ten females and eight males. Most had completed a bachelor's degree in Pharmacy. Their ages ranged from 29 to 72 years.

Results

The results are presented by the main themes that emerged during the data collection. These themes include communication quality, communication and imbalanced power and beliefs and instruction. The theme of communication quality emphasizes the dispensing behavior and communication between antibiotic dispensers and users. The second theme of communication and imbalanced power focuses on the space linked with the power to control antibiotics. Finally, the theme of beliefs and given instruction reveals the beliefs contradicted antibiotic instructions affecting antibiotic adherence.

Theme 1: Communication quality

This theme focuses on communication between dispensers (suppliers) and users (community members) and is mainly related to communication quality. Both dispensers and users have raised concerns of communication constraints, language use, and incomplete information.

Constraints to communication (i.e., comprehension) at different healthcare system levels seem to have similarities and differences. The major constraints to effective communication appeared at the tertiary, secondary, and (less so in the) primary care level in the public sector. By contrast, there were the least constraints at the private health facilities. The explanation may be that the more highly managed and bureaucratic procedures in the public healthcare setting are more complex and confusing for the client than the more service oriented system in the private setting.

In the public tertiary care setting, the interaction between dispensers and users is constrained by time limitations. This is primarily caused by overcrowding, especially when doctors initiate physical exams simultaneously, and prescriptions are concurrently sent to the pharmacy section. As a result, the pharmacy section becomes overwhelmed by a high volume of medical orders, leading to a scarcity of time for providing instructions.

> *"... in the late morning, when the patients accumulate, there are long queues, and we have to talk fast. Some patients can understand the information, but others may not."*
>
> (IDI, supplier, female, aged 40+, public hospital)

The communication constraints observed at the tertiary care level are also prevalent at the public secondary care level. Additionally, our participant noted that communication challenges are not solely attributed to the healthcare system but are influenced by factors such as patients' time and surrounding environment.

> *"There are many factors (affecting the difficulty in communication) such as when the patients were in a hurry or there are too many patients…moreover, it is noisy from the megaphone (calling the ''client's' name)."*
>
> (IDI, supplier, female, aged 40+, public hospital)

At the primary care level, the atmosphere is generally more laid-back, allowing staff to dedicate ample time to interacting with patients. Nonetheless, a challenge arises when communicating with specific groups, such as cross-border migrants who are not of Thai origin. This difficulty arises from their limited understanding of the Thai dialect.

> *"Now we have problems in communicating with migrants from Myanmar. Some can speak Thai a little bit, but some cannot speak at all. Often, there is a supervisor from the workplace of the migrants to serve as translator and advocate. However, we are not sure whether the clients will follow our instructions on proper use of the drugs".*
>
> (IDI, supplier, female, aged 40+, public hospital)

In contrast, the private healthcare setting offered greater flexibility in terms of time for patient communication, providing ample opportunity for interaction between healthcare providers and the individuals seeking care. The dispenser employs various strategies at this facility to ensure patient attentiveness toward the provided instructions.

> *"We normally give direct information to the patients. Apart from that, we observe how they respond to our explanation to assess comprehension. When we complete the instructions, we will ask whether they understand how to take the drugs. We also use some techniques to focus their attention, such as asking what types of medicines they are allergic to, or addressing them by their name"*
>
> (IDI, supplier, male, aged 50+, private hospital)

The community members discussed that they were given information about antibiotics related to their illness but did not fully understand the explanation. Some felt that the information was insufficient and wanted to know more about antibiotics.

"When talking about germ killer medicine, we only take it because they told us to, without any explanation. They only tell us that this is a germ killer medicine, but do not explain what types of germs it kills"."

(FGD community members, male, aged 60+, urban).

Apart from time constraints that limit the received-information, language use is another

Can you please provide an example of such a tecnical term?

issue, raised by both the suppliers and community members. The technical term that uses by dispensers but the users have no idea about such term. This used term sometimes lead confusion among community members. Some ~~of~~ community members mentioned that sometimes, they were given an explanation why they had to finish all the antibiotics, but some of the community members could not understand the term "antibiotic resistance".

"I do not know. I heard them say 'drug 'resistance'. I do not know what they are referring to"

(IDI community members, female, aged 47, Semi-urban)

The community members also needed simple words to understand the content rather than the technical term that they rarely understood. The community members from the FGD mentioned that the technical term confused them, making it hard for them to follow the content.

"The providers need simple words that the villagers are familiar with; then they will understand....I used to invite a nurse, who communicated by using simple words...not the technical terms during a meeting"

(FDG, community members, female, aged 15-59, semi-urban).

Although time constraints were different between the levels of the healthcare service system, the interpretation of information on antibiotics seemed to be similar. This reflects that

the dispensers are familiar with such information. Imbalance of information about antibiotics between dispensers and users occurred. Results in most users receiving or retaining imperfect information about the use of antibiotics.

> *"We need to ask the pharmacist how to take the tablets. If there is more than one pill, do I take them together or at different times? I get confused".*
>
> (FGD community members, female, aged 15-59, semi-urban)

While the importance of providing instructions for antibiotic use was acknowledged, our participants noted that when antibiotics are obtained from grocery stores, there are no accompanying instructions. In this scenario, users simply inform the seller of the specific antibiotic they wish to purchase.

> *"They (sellers at grocery shops)do not tell anything about the medicine we bought"*
>
> (FGD community members, female aged 18-30, semi-urban)

Theme 2: Communication and Imbalanced power

This theme focuses on the relationship between dispensers and users regarding the access and utilization of antibiotics. The problem of accessing antibiotics is closely tied to the choice between public and private healthcare facilities, while the location of administration influences the issue of their usage, whether it occurs at the forefront (health facility) or in the background (home). These concerns have been brought up for consideration.

Although they have a health scheme that provides them with affordable or free healthcare services, numerous Thai still pay out of pocket when the prescribed medication was not what they expected. As a result, they opt for private sector services, thereby incurring the entire expense. Some suppliers acknowledge that community members are entitled to free access to medication, yet some individuals even opt for informal alternatives when they are unable to obtain the desired medication.

"We have concerns about the 'clients' health scheme, some of them can get service with no cost. Anyway some choose to buy medicines from drugstores. If they do not feel better, they come to see us. If they ask us for a specific medicine which we refuse to dispense, they say that they will just go buy it from the drugstore"

(IDI, supplier, female, aged 40+, public hospital)

Community members have additionally conveyed that in instances where they are unable to obtain antibiotics from public health facilities, they resort to purchasing them from local drugstores.

"Once I went to see the doctor at the hospital and I did not get antibiotics, I decided to buy Amoxicillin from the drug store because I know that I had tonsillitis"

(FGD community members, elderly, aged 60+, semi-urban)

Access to antibiotics reflects how power is exercised. At public healthcare facilities, the users have less power to negotiate with dispensers. In other words, they let the healthcare providers be a legitimate interpreter in order to dispense antibiotics to them.

"The doctor told me that this medicine is a 'germ killer." I can understand easily that taking the germ killer will relieve my sickness quickly.....I never doubted the doctor; I just followed his instruction on how to take the medicine after or before meals. I did not have knowledge on this, so I did whatever they told me to do. However, if I take the medicine and I am not cured, I will buy new medicines from another source"

(IDI community members, male, aged 45, semi-urban).

By contrast, the clients have some power to bargain when they are at private health outlets. Private health facility is more patient-oriented, which allows the users to exercise power. For instance, they can ask for medicine if they feel they have another choice.

"Some clients, they are not happy with amoxicillin, they asked for Augmentin. We need some times to explain to them why the doctor starts with amoxicillin. Some clients are still not satisfied with our explanation, in this case, we have to consult the doctor".

(IDI supplier, male aged 50+, private hospital, urban)

Overall, however, it seems that users have the most power at the unlicensed shops that sell antibiotics. Here, they generally get what they demand. Our participants mentioned that sometimes community members just named the medicine they wanted, and these included antibiotics.

"At the drug store, they will buy these medicines (T-C mycin, penicillin (500,000), ampicillin) for wound and sore throat."

(FGD community members, female, aged 18-59, semi-urban)

Although the users have less power at the front space (health facility), some users gain control of antibiotic use when they take matters into their own hands. After getting antibiotics, some community members reported that the use of antibiotics depends on their own decision once the antibiotics are with them.

"We got instructions, but sometimes we did not follow them. It depends on ourselves because we are already feeling better"

(IDI community members, male, aged 60+ years old, semi-urban area)

Theme 3: Beliefs and antibiotic instruction

The main focus is on the contradiction between people's beliefs and the recommended antibiotic guidelines, leading to a lack of adherence. Antibiotic practices are influenced by

individuals' beliefs, regardless of whether they choose to follow the instructions provided. This directly affects antibiotic adherence.

Dispensers

Dispensers at formal healthcare facilities, whether public or private, always provide instructions in both oral and written formats to patients or users. Dispenser mostly inform the community member to complete course of antibiotic.

> *"For antibiotics, some customers specify their names. I advise them to finish the entire pack; they must take the full course. Some people ask how many days they have to take it. I dispense one pack at a time and advise them to finish it all. If the symptoms are still not completely resolved, then I ask them to come back and receive more medication. Typically, I dispense at least 3 days' worth of amoxicillin and 5 days' worth of antibiotics for patients with urinary tract infections."*
>
> *"On the medication bag, I provide the written instruction (i.e., medication for infection and advise taking all of them)."*

(IDI supplier, female, aged 42, pharmacy shop, semi-urban)

Community members have verified that the provided information is consistent in both oral and written forms.

> *"Actually, the pharmacist told me ~~that~~ how to take the medicine and also mentioned in the written instruction how many times a day and before or after meal"*

(FGD community members, elderly, aged 60+, semi-urban)

Write "the vast majority of the users" instead, if this doesn't apply to all the users (see below)?

However, the users stopped taking antibiotics once they experienced relief. In the FGD with village health volunteers, it was a surprise that among even these trained volunteers, who know that they have to complete the full dose of antibiotics in whatever conditions, only two in seven participants said that they took the complete dose of the antibiotics when they were ill. The others indicated that they stop the antibiotics when they feel better. This shows how ingrained this practice is at the village level.

Participant 1: "I stop, when I feel better"

Participant 2: "I took it for only 2 days"

Participant 3: "When I feel a little bit better, I stop"

Participant 4: "Somebody said the antibiotic is dangerous, I 'didn't dare to take a lot of it"

(FGD, community members, female village health volunteers, urban)

In addition to the village health volunteers' views, community members also confirmed their tendency to prematurely discontinue antibiotic usage when they feel improvement. Information gathered from 7 Focus Group Discussions (FGDs) and 9 In-Depth Interviews (IDIs) with community members confirmed that many stopped taking antibiotics as instructed.

"I think only 20 percent of those community members finish all antibiotics as indicated in the instruction"

(FGD community members, female aged 18-59, semi-urban)

After getting antibiotic instructions, whether the community members want to follow the instructions or not is sometimes linked to their level of knowledge. One of the dispensers indicated that knowledge about antibiotics would help in adherence.

"They have the knowledge, but each individual has a different level of knowledge. Those who care about their health will seek accurate information and take the antibiotic properly"

(IDI supplier, female, aged 42, urban)

Although most community members believe in the curative powers of antibiotics for their illnesses, many people try to avoid taking them. This is due to the fact that they fear the side effects and interaction with other medications they take. Thus, they try to take less or stop taking antibiotics as soon as possible and limit overall antibiotic usage.

Q: From the instruction to finish all of them (antibiotics), some of you told me you stopped when you felt better. Do any of you do the same?

Participant 6: Yes, when I got rid of sick, I stopped. I am afraid that it may affect the drug I take daily.

(FGD community members, female aged 30+, urban)

The dispenser also corroborated these fears, stating that some community members are apprehensive about antibiotics' side effects and specifically request fewer such medicines. For instance, one individual expressed concern that antibiotics might harm their kidneys and liver, leading them to request only two antibiotic pills from the pharmacy.

"He told me that he was afraid of it (antibiotics*) because it is going to destroy his kidneys and liver. He was really afraid and asked me for only two antibiotic pills"*

(IDI supplier, female, aged 33, pharmacy shop, urban)

Discussion

This study attempts to understand the dispenser and users' communication, allowing the researcher to find interaction between the two key actors . Understanding antibiotic dispensing and usage with regard to communication is important to design and implement appropriate interventions to address the issue in the broader public health interest.

Our findings point out that constraints in communication occur due to overcrowding and rushed processing of a large number of patients, limiting time for client education. This situation is particularly prevalent in public tertiary and secondary care settings. Additionally, a lack of communication skills is a significant deficit among some dispensers. These factors may lead to imperfect information exchange and confusion about the indications for antibiotic use. Previous studies found that a shortage of consulting time and poor communication skills meant inadequate explanations for patients and inappropriate antibiotic use [27-29]. The language used in communicating with clients is another concern of our study since it was

found that users were less likely to understand the dispenser's language in certain situations. This can lead to misunderstanding about antibiotic use. A previous study suggested that plain and simple language is necessary for effective communication about antibiotics [48]. Furthermore, we shoud consider specific groups, such as migrants with limited proficiency in

The reader doesn’t know much about the rural population, do people generally own a smart phone and can afford mobile data in rural areas?

the Thai dialect. To address this issue, we propose the use of QR codes containing online information presented in pictograms or the languages spoken by migrants. This approach can

add spacing before reference (49)

effectively convey medication information and enhance comprehension[49]

One of the important findings in our study is that the term "antibiotic resistance" is almost universally misunderstood or unknown among people at the village level. This finding confirms the results of a previous study in Thailand which found that community residents were not familiar with the term "antibiotic resistance". This may be due to the fact that they never heard, never believed, and/or never feared drug resistance [50]. Thus, our findings suggest that it is important to address this issue by providing training or conducting workshops for dispensers to enhance communication skills, particularly communication using lay language. Additionally, integrating QR codes with easily understandable pictogram-based online information, a strategy proven effective in previous research [49], may help increase antibiotic compliance and overall satisfaction of the users.

Another concern of our study is the practice of buying antibiotics for self-medication for mild illness from grocery stores without instructions. Medication from these shops are mostly dispensed by non-qualified dispensers, ~~even though~~. It reveals that the community

Please rephrase this sentence (line 512)

members are easy to get antibiotics from non-qualified dispensers in the study areas. A previous study found that non-qualified dispensers significantly aggravate the inappropriate use of antibiotics in the community setting [51]. The previous study also found that patients who received antibiotics without a prescription were more likely to stop when their symptoms improved compared to those with a prescription [52]. However, Thailand has a national drug

law that allows antibiotics to be dispensed by doctors, pharmacists, and health authorities. Ironically, the fact that antibiotics can be bought over the counter without a prescription means that non-qualified sellers can appear qualified to the uninformed user. Thus, antibiotics are distributed through several channels [53], including legal and illegal distributors [54]. Therefore, a national strategy aiming to improve antibiotics dispensing and reduce non-essential and incomplete use of the drugs also needs to focus on private health facilities, particularly those with unqualified dispensers. This could be one of the effective measures for controlling bacterial resistance**.** In addition, the finding reinforces that effective instruction, law enforcement and prescription are critical in managing antibiotics in the community setting.

”A” previous study… add a comma (after the word antibiotics line 527)

Previous study pointed out that promoting responsible use of antibiotics necessitates acknowledging the significance of power dynamics and autonomy within the healthcare

How do you define ”power”?

system [26]. Thus, imbalanced power in communication is another concern of our study. The power in our study also refers to the interaction between dispensers and users at front space (health facility) and back space (home) settings, through access to and knowledge of

Please specify what literature you refer to here (add reference on line 533)

antibiotics. Literature reveals that access to power and resources is negotiated in everyday life. Thus, the health facility setting is not only a clinical space but also a social space in which unequal power relations occur [55]. There is a power imbalance between healthcare providers and clients, particularly in healthcare settings where patients are most likely to be subordinated [27, 30]. Our study found different levels of imbalance of power between

Please examplify this power imbalance – how is it visible?

antibiotic dispensers and users. At ~~the tertiary and secondary~~ care levels, such an imbalance seems stronger than at the primary and private healthcare levels. The imbalance of power is less in the private sector due to the fact that they are more customer-oriented and less bureaucratic. Notably, the power advantage shifts to the user interacting with the unlicensed dispensers. In highly regulated and managed healthcare settings such as tertiary and

secondary care outlets, users may feel a socio-cultural pressure to behave as "good patients" in the eyes of dispensers. In that space, the 'user's role is passive and compliant, and the client is uncomfortable to speak up [27, 56, 57]. Conversely, when the balance of power is more

Please change the word "level" here, perhaps you can use "even" instead?

level, the user will feel more confident in speaking up. Not only access to but also knowledge of antibiotics ~~also~~ creates a power imbalance. Our study found that users do not necessarily understand the exact nature of their condition, and may not understand their treatment options. Thus they defer their decisions to the dispensers as the legitimated interpreter for their illness.

In contrast, in the back space of control over antibiotic use, community members have full power to control use. Exercising this control with only limited knowledge of antibiotics may lead to the inappropriate use of such powerful medicines. In addition, lacking knowledge of antibiotics may be a symptom of a more significant health illiteracy problem in parts of society. Health literacy recognizes the ability to understand and critically evaluate health information and make health-related decisions [58]. Previous studies in other parts of the world also pointed out that education and health literacy played a crucial role in the reduction of inappropriate use of antibiotics [20, 59-63]. Thus, our findings call for enhancing health literacy, which may influence the dispenser-user relationship, self-care, and the use of antibiotics.

Our finding reveal that the most important instruction for antibiotic use includes how to take the drugs and the need to finish them all. But users are not always told the reason why they have to finish the drugs, especially if they feel cured after a day or two. This belief discourages compliance and is a primary reason for the lack of adherence.The contradiction between beliefs and antibiotic instruction, while giving instructions for antibiotic use, operates through the prevailing beliefs of the community members. This interaction can cause

stopping or continuing antibiotics due to misconception about antibiotics and fear of side effects, which, in turn, reflects treatment compliance.

This study found that participants universally received instructions for antibiotics use from formal dispensers, while those who got their supply from unlicensed sellers did not receive instructions. The main information from the formal sector included how to take the drugs and the need to finish the complete dose, regardless of how well they felt. Notably, users are not always told the reason why they have to finish the drugs, especially if they feel cured after a day or two. This belief discourages compliance and is a primary reason for the lack of adherence. Furthermore, it appears that some community members seem to not be fully accepted these clear instructions for antibiotic use because they fear of the side effect. This fear of side effects can lead individuals to prematurely discontinue or modify their antibiotic treatment, even if it contradicts the recommended instructions. A previous study found that misunderstanding of the consequences of unnecessary antibiotic use brought about non-adherence [64].

This sentence needs some rephrasing

In addition to misconceptions, fear also leads individuals to prematurely stop or modify antibiotic treatment, despite contradicting the recommended instructions. A study in Saudi Arabia demonstrated that participants would take less of their antibiotics to avoid adverse effects without consulting a healthcare professional[37]. Our finding sheds light on the significance of addressing beliefs and misunderstandings surrounding antibiotic use to improve adherence rates in the community. This highlights the need for comprehensive education and communication efforts to ensure that individuals understand the rationale behind antibiotic treatment guidelines and feel more confident in adhering to them, especially regarding when and how to take antibiotics. For instance, taking antibiotics as prescribed and knowing when not to use them, particularly for viral infections[65].

Strengths and limitations

The strength of this study is the integration of dispensers and users. Healthcare providers, suppliers, and users of antibiotics provided practical insights on how and why these drugs are controlled and dispensed, how users are instructed, and how clients use the drugs.

This study also has limitations. Since dispensing antibiotics is regulated by the national drug law, our study was not able to obtain empirical information from illegal antibiotic dispensers in the act of selling and dispensing by unqualified persons. This limitation calls for further research that deals with those illegal dispensers, and perhaps indigenous field worker sampling (IFWS) is required because it allows local community members ~~member~~ who has the advantage of reaching the target sample to be trained as investigators ~~investigator~~[66].

Conclusions

The evolving practices of antibiotic usage depend on both dispensers and users and their communication. Future interventions should aim to enhance communication between dispensers and users, improve users' knowledge, and provide clear instructions to facilitate user compliance. Exploring alternative communication channels, such as QR codes, is a viable option. This approach can contribute to reducing one of the major causes of antimicrobial resistance (AMR).

Suppoting information

S1 File. Research tool.doxc

S2 File. Healthcare delivery system in Thailand.doxc

Competing interests

The authors declare that they have no competing interests.

Author contributions

The main contributor to this study was MS who was responsible for conception, software, visualization, formal data analysis and writing the original draft with the support from all authors. Data curation and investigation were carried out by MS, SP and WJ. MS,SP,WJ and HW contributed to the methodology. MS,SP,WJ,HW reviewed the manuscript. Checking validation of codes, interpretation, and administered the project had been done by MS, SP, and WJ. SP and HW involved in funding acquisition. All authors read and approved the final manuscript.

Acknowledgments

The kind cooperation and sharing of information of all participants made this study possible. We would like to thank all of them. We would also like to thank Jettapon Sangkla , who assisted us with the data collection. Finally, we would like to express our appreciation to Osman Sankoh, Margaret Gyapong, Johannes John-Langba,and John Kinsman who supported the study.

Funding

The Wellcome Trust , UK funded the study. The Institute for Population and Social Research, Mahidol University, Thailand, supported data analysis and manuscript writing.

Availability of data and materials

All relevant data are within the manuscript and its Supporting Information files.

Consent for publication

All authors have approved the manuscript and take responsibility for its accuracy and integrity of the work.

References

1. World Health Organization. Prioritization of pathogens to guide discovery, research and development of new antibiotics for drug-resistant bacterial infections, including tuberculosis. World Health Organization, 2017.
2. Tangcharoensathien V, Sommanustweechai A, Chanvatik S, Kosiyaporn H, Tisocki K. Addressing the threat of antibiotic resistance in Thailand: monitoring population knowledge and awareness. WHO South-East Asia journal of public health. 2018;7(2):73-8. Epub 2018/08/24. doi: 10.4103/2224-3151.239417. PubMed PMID: 30136664.
3. Thamlikitkul V. STOP antimicrobial resistance: everybody's business. Siriraj Medical Journal. 2017;66(6):240-6.
4. Sumpradit N, Chongtrakul P, Anuwong K, Pumtong S, Kongsomboon K, Butdeemee P, et al. Antibiotics Smart Use: a workable model for promoting the rational use of medicines in Thailand. Bulletin of the World Health Organization. 2012;90:905-13.
5. Sumpradit N, Wongkongkathep S, Poonpolsup S, Janejai N, Paveenkittiporn W, Boonyarit P, et al. New chapter in tackling antimicrobial resistance in Thailand. BMJ. 2017;358:j3415.
6. Boonyasiri A, Thamlikitkul V. Effectiveness of multifaceted interventions on rational use of antibiotics for patients with upper respiratory tract infections and acute diarrhea. Journal of the Medical Association of Thailand = Chotmaihet thangphaet. 2014;97 Suppl 3:S13-9. Epub 2014/04/30. PubMed PMID: 24772576.
7. Phueanpinit P, Siriwattanasakul N, Tichakorn P, Klanbut S. Knowledge and perceptions of outpatients regarding upper respiratory tract infections and awareness of an Antibiotics Smart Use campaign in Thailand. Journal of Pharmacy Practice and Research. 2022;52(4):294-302.
8. Tangcharoensathien V, Chanvatik S, Kosiyaporn H, Kirivan S, Kaewkhankhaeng W, Thunyahan A, et al. Population knowledge and awareness of antibiotic use and antimicrobial resistance: results from national household survey 2019 and changes from 2017. BMC Public Health. 2021;21(1):2188. doi: 10.1186/s12889-021-12237-y.

9. Lopez□Vazquez P, Vazquez□Lago JM, Figueiras A. Misprescription of antibiotics in primary care: a critical systematic review of its determinants. Journal of Evaluation in Clinical Practice. 2012;18(2):473-84.

10. Goossens H, Ferech M, Vander Stichele R, Elseviers M. Outpatient antibiotic use in Europe and association with resistance: a cross-national database study. The Lancet. 2005;365(9459):579-87. doi: https://doi.org/10.1016/S0140-6736(05)17907-0.

11. Tenover FC. Mechanisms of antimicrobial resistance in bacteria. American Journal of Infection Control. 2006;34(5, Supplement):S3-S10. doi: https://doi.org/10.1016/j.ajic.2006.05.219.

12. Awad AI, Aboud EA. Knowledge, attitude and practice towards antibiotic use among the public in Kuwait. PloS ONE. 2015;10(2):e0117910-e. doi: 10.1371/journal.pone.0117910. PubMed PMID: 25675405.

13. Messano GA, Cancrini F, Marsella LT. Antibiotic resistance and defensive medicine, a modern challenge for an old problem: the case of Ureaplasma urealyticum. Igiene e Sanita Pubblica. 2014;70(3):295-304. Epub 2014/09/07. PubMed PMID: 25194120.

14. Nicholson A, Tennant I, White L, Thoms-Rodriguez C-A, Cook L, Johnson S, et al. The knowledge, attitudes and practices of doctors regarding antibiotic resistance at a tertiary care institution in the Caribbean. Antimicrobial Resistance & Infection Control. 2018;7(1):23. doi: 10.1186/s13756-018-0315-3.

15. Vaz LE, Kleinman KP, Lakoma MD, Dutta-Linn MM, Nahill C, Hellinger J, et al. Prevalence of parental misconceptions about antibiotic use. Pediatrics. 2015:peds. 2015-0883.

16. Norris P, Chamberlain K, Dew K, Gabe J, Hodgetts D, Madden H. Public beliefs about antibiotics, infection and resistance: a qualitative study. Antibiotics. 2013;2(4):465-76.

17. Thaler RH, Sunstein CR. Nudge: Improving decisions about health, wealth, and happiness: Penguin; 2009.

18. The McDonnell Norms Group. Antibiotic overuse: the influence of social norms. Journal of the American College of Surgeons. 2008;207(2):265-75.

19. Touboul-Lundgren P, Jensen S, Drai J, Lindbæk M. Identification of cultural determinants of antibiotic use cited in primary care in Europe: a mixed research synthesis study of integrated design "Culture is all around us". BMC Public Health. 2015;15(1):908. doi: 10.1186/s12889-015-2254-8.

20. Widayati A, Suryawati S, de Crespigny C, Hiller JE. Knowledge and beliefs about antibiotics among people in Yogyakarta City Indonesia: a cross sectional population-based survey. Antimicrobial Resistance and Infection Control. 2012;1(1):38. doi: 10.1186/2047-2994-1-38.

21. Ratna H. The Importance of Effective Communication in Healthcare Practice. Harvard Public Health Review. 2019;23:1-6.

22. Coxeter P, Del Mar CB, McGregor L, Beller EM, Hoffmann TC. Interventions to facilitate shared decision making to address antibiotic use for acute respiratory infections in primary care. Cochrane Database of Systematic Reviews. 2015;(11).

23. Mathew P, Sivaraman S, Chandy S. Communication strategies for improving public awareness on appropriate antibiotic use: Bridging a vital gap for action on antibiotic resistance. Journal of Family Medicine and Primary Care. 2019;8(6):1867-71. Epub 2019/07/25. doi: 10.4103/jfmpc.jfmpc□263□19. PubMed PMID: 31334147; PubMed Central PMCID: PMCPMC6618197.

24. Sun G, Manzanares K, Foley KA, Zhou Y, MacGeorge EL. Antibiotic stewardship with upper respiratory tract infection patients at student health centers: Providers' communication experiences and strategies. American Journal of Infection Control. 2023;51(2):154-8. doi: https://doi.org/10.1016/j.ajic.2022.05.013.

25. Bell BG, Schellevis F, Stobberingh E, Goossens H, Pringle M. A systematic review and meta-analysis of the effects of antibiotic consumption on antibiotic resistance. BMC Infectious Diseases. 2014;14:13. Epub 2014/01/11. doi: 10.1186/1471-2334-14-13. PubMed PMID: 24405683; PubMed Central PMCID: PMCPMC3897982.

26. Bianco A, Licata F, Trovato A, Napolitano F, Pavia M. Antibiotic-Dispensing Practice in Community Pharmacies: Results of a Cross-Sectional Study in Italy. Antimicrobial Agents and Chemotherapy. 2021;65(6). Epub 2021/03/31. doi: 10.1128/aac.02729-20. PubMed PMID: 33781998; PubMed Central PMCID: PMCPMC8316047.

27. Frosch DL, May SG, Rendle KA, Tietbohl C, Elwyn G. Authoritarian physicians and patients' fear of being labeled 'difficult'among key obstacles to shared decision making. Health affairs. 2012;31(5):1030-8.

28. Kumar S, Little P, Britten N. Why do general practitioners prescribe antibiotics for sore throat? Grounded theory interview study. BMJ. 2003;326(7381):138. doi: 10.1136/bmj.326.7381.138.

29. Lorenzetti RC, Jacques CM, Donovan C, Cottrell S, Buck J. Managing difficult encounters: understanding physician, patient, and situational factors. American family physician. 2013;87(6).

30. Scholz B, Bocking J, Happell B. Breaking through the Glass Ceiling: Consumers in Mental Health Organisations' Hierarchies. Issues in Mental Health Nursing. 2017;38(5):374-80. doi: 10.1080/01612840.2017.1280106.

31. Gunasekera YD, Kinnison T, Kottawatta SA, Silva-Fletcher A, Kalupahana RS. Misconceptions of Antibiotics as a Potential Explanation for Their Misuse. A Survey of the General Public in a Rural and Urban Community in Sri Lanka. Antibiotics. 2022;11(4). Epub 2022/04/24. doi: 10.3390/antibiotics11040454. PubMed PMID: 35453206; PubMed Central PMCID: PMCPMC9024968.

32. Afari-Asiedu S, Hulscher M, Abdulai MA, Boamah-Kaali E, Asante KP, Wertheim HFL. Every medicine is medicine; exploring inappropriate antibiotic use at the community level in rural Ghana. BMC Public Health. 2020;20(1):1103. doi: 10.1186/s12889-020-09204-4.

33. Alkhuzaei A, Salama R, Eljak IE, Chehab MAH, Selim NAA. The knowledge, practice, and attitude regarding antibiotic use among attendants of primary health care centres: A cross-sectional study in Qatar. International Journal of Community Medicine and Public Health. 2017;4(11):3969.

34. Bagshaw SM, Kellner JD. Beliefs and behaviours of parents regarding antibiotic use by children. The Canadian Journal of Infectious Diseases = Journal Canadien des Maladies Infectieuses. 2001;12(2):93-7. PubMed PMID: 18159323.

35. Do NT, Vu HT, Nguyen CT, Punpuing S, Khan WA, Gyapong M, et al. Community-based antibiotic access and use in six low-income and middle-income countries: a mixed-method approach. The Lancet Global Health. 2021;9(5):e610-e9.

36. Otaigbe II, Elikwu CJ. Drivers of inappropriate antibiotic use in low- and middle-income countries. JAC-Antimicrobial Resistance. 2023;5(3). doi: 10.1093/jacamr/dlad062.

37. Alhomoud F, Aljamea Z, Basalelah L. "Antibiotics kill things very quickly" - consumers' perspectives on non-prescribed antibiotic use in Saudi Arabia. BMC Public Health. 2018;18(1):1177. doi: 10.1186/s12889-018-6088-z.

38. Gaygısız Ü, Lajunen T, Gaygısız E. Community Use of Antibiotics in Turkey: The Role of Knowledge, Beliefs, Attitudes, and Health Anxiety. Antibiotics. 2021;10(10). Epub 2021/10/24. doi: 10.3390/antibiotics10101171. PubMed PMID: 34680752; PubMed Central PMCID: PMCPMC8532753.

39. Cross ELA, Tolfree R, Kipping R. Systematic review of public-targeted communication interventions to improve antibiotic use. Journal of Antimicrobial Chemotherapy. 2016;72(4):975-87. doi: 10.1093/jac/dkw520.

40. Arparsrithongsagul S, Kulsomboon V, Zuckerman IH. Multidisciplinary perspective intervention with community involvement to decrease antibiotic sales in village groceries in Thailand. Asia-Pacific Journal of Public Health. 2015;27(2):Np2480-8. Epub 2013/03/29. doi: 10.1177/1010539513479968. PubMed PMID: 23536236.

41. Charoenboon N, Haenssgen MJ, Warapikuptanun P, Xayavong T, Khine Zaw Y. Translating antimicrobial resistance: a case study of context and consequences of antibiotic-related communication in three northern Thai villages. Palgrave Communications. 2019;5(1):1-24.

42. Altiner A, Brockmann S, Sielk M, Wilm S, Wegscheider K, Abholz H-H. Reducing antibiotic prescriptions for acute cough by motivating GPs to change their attitudes to communication and empowering patients: a cluster-randomized intervention study. Journal of Antimicrobial Chemotherapy. 2007;60(3):638-44.

43. Mangione-Smith R, McGlynn EA, Elliott MN, Krogstad P, Brook RH. The relationship between perceived parental expectations and pediatrician antimicrobial prescribing behavior. Pediatrics. 1999;103(4):711-8.

44. Wertheim HF, Chuc NTK, Punpuing S, Khan WA, Gyapong M, Asante KP, et al. Community-level antibiotic access and use (ABACUS) in low-and middle-income countries: finding targets for

social interventions to improve appropriate antimicrobial use–an observational multi-Centre study. Wellcome Open Research. 2017;2.

45. Institute for Population and Social Research Mahidol University. Report of Round 5 Census (2004):Kanchanaburi Project Nakhon Pathom: Institute for Population and Social Research, Mahidol university, 2007.

46. ICT Services and System Development and Division of Epidemiology and Global Health. OpenCode 4.0. Umeå: University of Umeå, Sweden; 2015.

47. Tong A, Sainsbury P, Craig J. Consolidated criteria for reporting qualitative research (COREQ): a 32-item checklist for interviews and focus groups. International journal for quality in health care. 2007;19(6):349-57.

48. Friedman D, Kanwat C, Headrick M, Patterson N, Neely J, Smith L. Importance of prudent antibiotic use on dairy farms in South Carolina: a pilot project on farmers' knowledge, attitudes and practices. Zoonoses and Public Health. 2007;54(9□10):366-75.

49. Dowse R. Pharmacists, are words enough? The case for pictograms as a valuable communication tool. Research in Social and Administrative Pharmacy. 2021;17(8):1518-22. doi: https://doi.org/10.1016/j.sapharm.2020.10.013.

50. Pharm SKB, Pharm YNB, Pharm SBB, Rattanaumpawan P, Boonyasiri A, Thamlikitkul V. Epidemiology of antibiotic use and antimicrobial resistance in selected communities in Thailand. Journal of the Medical Association of Thailand = Chotmaihet thangphaet. 2016;99(3):270-5.

51. Ocan M, Obuku EA, Bwanga F, Akena D, Richard S, Ogwal-Okeng J, et al. Household antimicrobial self-medication: a systematic review and meta-analysis of the burden, risk factors and outcomes in developing countries. BMC Public Health. 2015;15:742. Epub 2015/08/02. doi: 10.1186/s12889-015-2109-3. PubMed PMID: 26231758; PubMed Central PMCID: PMCPMC4522083.

52. Jamhour A, El-Kheir A, Salameh P, Hanna PA, Mansour H. Antibiotic knowledge and self-medication practices in a developing country: A cross-sectional study. American Journal of Infection Control. 2017;45(4):384-8. doi: https://doi.org/10.1016/j.ajic.2016.11.026.

53. Sommanustweechai A, Chanvatik S, Sermsinsiri V, Sivilaikul S, Patcharanarumol W, Yeung S, et al. Antibiotic distribution channels in Thailand: results of key-informant interviews, reviews of drug regulations and database searches. Bulletin of the World Health Organization. 2018;96(2):101-9. Epub 2018/01/02. doi: 10.2471/BLT.17.199679. PubMed PMID: 29403113.

54. Holloway KA, Kotwani A, Batmanabane G, Puri M, Tisocki K. Antibiotic use in South East Asia and policies to promote appropriate use: reports from country situational analyses. BMJ. 2017;358:j2291. doi: 10.1136/bmj.j2291.

55. Tang SY, Browne AJ, Mussell B, Smye VL, Rodney P. 'Underclassism' and access to healthcare in urban centres. Sociology of Health & Illness. 2015;37(5):698-714. Epub 2015/02/28. doi: 10.1111/1467-9566.12236. PubMed PMID: 25720520.

56. Francis R. Report of the Mid Staffordshire NHS Foundation Trust public inquiry: executive summary: The Stationery Office; 2013.

57. Joseph-Williams N, Edwards A, Elwyn G. Power imbalance prevents shared decision making. BMJ : British Medical Journal. 2014;348:g3178. doi: 10.1136/bmj.g3178.

58. Health literacy. The solid facts [Internet]. World Health Organization. 2013 [cited 19 June 2019]. Available from: http://publichealthwell.ie/node/534072.

59. Alhomoud F, Aljamea Z, Almahasnah R, Alkhalifah K, Basalelah L, Alhomoud FK. Self-medication and self-prescription with antibiotics in the Middle East—do they really happen? A systematic review of the prevalence, possible reasons, and outcomes. International Journal of Infectious Diseases. 2017;57:3-12. doi: https://doi.org/10.1016/j.ijid.2017.01.014.

60. Barker AK, Brown K, Ahsan M, Sengupta S, Safdar N. Social determinants of antibiotic misuse: a qualitative study of community members in Haryana, India. BMC Public Health. 2017;17(1):333-. doi: 10.1186/s12889-017-4261-4. PubMed PMID: 28420365.

61. Céspedes A, Larson E. Knowledge, attitudes, and practices regarding antibiotic use among Latinos in the United States: Review and recommendations. American Journal of Infection Control. 2006;34(8):495-502. doi: https://doi.org/10.1016/j.ajic.2006.01.005.

62. Salm F, Ernsting C, Kuhlmey A, Kanzler M, Gastmeier P, Gellert P. Antibiotic use, knowledge and health literacy among the general population in Berlin, Germany and its surrounding rural areas. PloS ONE. 2018;13(2):e0193336-e. doi: 10.1371/journal.pone.0193336. PubMed PMID: 29474470.

63. Shehadeh M, Suaifan G, Darwish RM, Wazaify M, Zaru L, Alja'fari S. Knowledge, attitudes and behavior regarding antibiotics use and misuse among adults in the community of Jordan. A pilot study. Saudi Pharmaceutical Journal. 2012;20(2):125-33. doi: https://doi.org/10.1016/j.jsps.2011.11.005.

64. Pinder R, Sallis A, Berry D, Chadborn T. Behaviour change and antibiotic prescribing in healthcare settings. Literature review and behavioural analysis. Public Health England. 2015.

65. Llewelyn MJ, Fitzpatrick JM, Darwin E, Gorton C, Paul J, Peto TE, et al. The antibiotic course has had its day. BMJ. 2017;358.

66. Shaghaghi A, Bhopal RS, Sheikh A. Approaches to Recruiting 'Hard-To-Reach' Populations into Re-search: A Review of the Literature. Health Promot Perspect. 2011;1(2):86-94. doi: 10.5681/hpp.2011.009. PubMed PMID: 24688904.

Click here to access/download
Supporting Information
S1 File-Research tools.docx

S2 File Healthcare delivery system in Thailand

Click here to access/download

Supporting Information

S2 File -Healthcare delivery system.docx
